# A morphometric, immunohistochemical, and in situ hybridization study of the dorsal raphe nucleus in major depression, bipolar disorder, schizophrenia, and suicide

**DOI:** 10.1016/j.jad.2011.10.043

**Published:** 2012-03

**Authors:** Paul R. Matthews, Paul J. Harrison

**Affiliations:** Department of Psychiatry, University of Oxford, Warneford Hospital, Oxford OX3 7JX, UK

**Keywords:** 5-HT1A receptor, HTR1A, Morphometry, Serotonin, Tryptophan hydroxylase

## Abstract

**Background:**

Several lines of evidence implicate 5-hydroxytryptamine (5-HT, serotonin) in the pathophysiology of mood disorders and suicide. However, it is unclear whether these conditions include morphological involvement of the dorsal raphe nucleus (DRN), the origin of most forebrain 5-HT innervation.

**Method:**

We used morphometric, immunohistochemical, and molecular methods to compare the DRN in post-mortem tissue of 50 subjects (13 controls, 14 major depressive disorder [MDD], 13 bipolar disorder, 10 schizophrenia; 17 of the cases died by suicide). NeuN and PH8 antibodies were used to assess all neurons and serotonergic neurons respectively; 5-HT_1A_ autoreceptor expression was investigated by regional and cellular in situ hybridization. Measurements were made at three rostrocaudal levels of the DRN.

**Results:**

In MDD, the area of the DRN was decreased. In bipolar disorder, serotonergic neuronal size was decreased. Suicide was associated with an increased DRN area, and with a higher density but decreased size of serotonergic neurons. Total neuronal density and 5-HT_1A_ receptor mRNA abundance were unaffected by diagnosis or suicide. No changes were seen in schizophrenia.

**Conclusion:**

The results show that mood disorders and suicide are associated with differential, limited morphological alterations of the DRN. The contrasting influences of MDD and suicide may explain some of the discrepancies between previous studies, since their design precluded detection of the effect.

## 1 Introduction

The 5-hydroxytryptamine (5-HT; serotonin) neurons that innervate the forebrain lie in the rostral raphe nuclei of the brainstem (Hornung, 2003; Molliver, 1987), with the dorsal raphe (DRN) being the largest nucleus (Baker et al., 1991). The DRN provides the main input to the frontal cortex (McQuade and Sharp, 1997) and an enlarged lateral subdivision characterises primates, including man (Hornung, 2003). In the DRN cells projecting to prefrontal cortex are preferentially found in more rostral, medial, and ventral subdivisions, and around half are non-serotonergic (Del Cid-Pellitero and Garzón, 2011; Wilson and Molliver, 1991). Afferent projections to the DRN are primarily from the limbic system (Hornung, 2003) but there is also a reciprocal innervation of the DRN from prefrontal cortex which modulates neuronal activity (Celada et al., 2001).

The 5-HT system has been implicated in many psychiatric disorders, including mood disorders (Barton et al., 2008; Deakin, 1998; Jans et al., 2007; Mahmood and Silverstone, 2001), and in suicide (Ernst et al., 2009; Mann et al., 1989; Placidi et al., 2001). The evidence is diverse, and includes alterations in 5-HT metabolism, 5-HT receptors and transporters, and associations with serotonergic gene polymorphisms. There is also evidence of decreased neuronal density and serotonergic abnormalities in prefrontal cortex of depressed suicides (Underwood et al., 2011).

In contrast, the morphology and cytoarchitecture of the DRN in these disorders have received limited attention. The existing studies have utilised conventional stains (Baumann et al., 2002; Underwood et al., 1999), or antibodies detecting tryptophan hydroxylase (TPH) to identify 5-HT neurons (Hendricksen et al., 2004; Syed et al., 2005; Underwood et al., 1999); there are also studies which have used other 5-HT markers (e.g. 5-HT_1A_ autoreceptors; Arango et al., 2001; Boldrini et al., 2008; Stockmeier et al., 1998), or a marker of raphe neuron ‘activation’ (Bielau et al., 2005). The studies have produced variable results, likely reflecting both methodological and clinical factors. For example, as well as measuring different parameters, and using various DRN sampling strategies, the studies are small, and differ in the subjects’ age, polarity of mood disorder, and presence of comorbid conditions.

This investigation was performed to help shed some further light on the involvement of the DRN in the neuropathology of mood disorder and suicide. It has a larger sample size than existing studies; it includes patients from three diagnostic groups (major depression [MDD], bipolar disorder, and schizophrenia, as well as suicides and non-suicides within each group), and uses three complementary techniques: NeuN to assess all neurons, and TPH immunohistochemistry and 5-HT_1A_R mRNA in situ hybridization (ISH) as markers of serotonergic neurons.

## 2 Materials and methods

### 2.1 Subject and tissue characteristics

Unfixed frozen 14 μm sections of brainstem were provided by the Stanley Neuropathology Consortium from their series of 60 subjects diagnosed (by DSM-IV criteria) with schizophrenia, bipolar disorder or MDD, and controls (Torrey et al., 2000). In each diagnostic group some subjects died by suicide. The sections provided were quite rostral, and tissue from ten subjects did not contain sufficient clearly discernible DRN to be included. Table 1 summarises the details of the resulting 50 subjects. Adjacent sections were taken for NeuN and PH8 immunostaining every 1 mm, and 1 section every 500 μm for 5-HT_1A_R ISH. The experiments described here, were carried out with ethical approval from Oxfordshire Research Ethics Committee B (#O02.040). All material was coded by the Stanley Medical Research Institute, and experiments and analyses conducted blind to diagnostic and other information.

### 2.2 Immunohistochemistry for NeuN and PH8

The NeuN antibody stains virtually all neuron populations and is widely used for morphometry (Mullen et al., 1992). It has the advantage over Nissl stains that glia are not labelled (Gittins and Harrison, 2004). Incubations were carried out at a concentration of 1:100 overnight at 4 °C. The PH8 antibody (originally raised against phenylalanine hydroxylase) has been successfully used to characterise the serotonergic system in human tissue (Tork et al., 1992), and our preliminary studies confirmed that the antibody did not label tyrosine hydroxylase in cells of the substantia nigra or locus coeruleus under these incubation (3 days at 4°; 1:500) and fixation conditions (4% paraformaldehyde for 5 min). Staining was performed using a secondary biotinylated antibody, avidin-biotin peroxidase complex, and 3,3′-diaminobenzidine. Omission of the primary antibody was used as a negative control. See Supplementary Methods for additional details of the immunohistochemical protocols.

### 2.3 Cell counting and morphometry

**Fig. 1:**
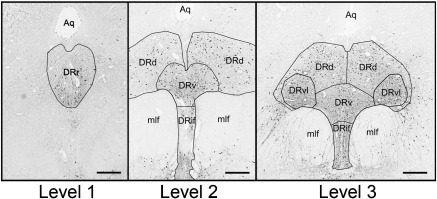
PH8 immunohistochemistry at the three rostrocaudal DRN ‘Levels’ defined in the text. All three images are from the same subject, in whom Level 2 is 1 mm caudal to Level 1, and Level 3 is 2 mm caudal to Level 2. Abbreviations: Aq: aquaduct; mlf: medial longitudinal fasciculus; DRr: rostral pole; DRd: dorsal subnucleus; DRv: ventral subnucleus; DRif: interfascicular subnucleus; DRvl: ventrolateral subnucleus. Scale bar: 1 mm.

Measurements were grouped by rostrocaudal level in the rostral DRN (Baker et al., 1990; Paxinos and Huang, 1995) as delineated by adjacent PH8 and NeuN-stained sections. Given the heterogeneity in the length and availability of the DRN, slides were assigned an anatomical level based upon features present in the section. Three levels were thus identified (Fig. 1). Level 1 is the rostral pole (DRr) of the DRN, at the level of the oculomotor nucleus; Level 2 lies between the caudal pole of the oculomotor nucleus and the rostral end of the trochlear nucleus and contains the dorsal (DRd), ventral (DRv) and interfascicular (DRif) subnuclei; at Level 3 the DRN is at its greatest width, with the ventrolateral subnucleus (DRvl) present in addition to the DRd, DRv and DRif.

PH8 staining was very dense and prevented resolution of the cell nucleus, so neuronal profiles were counted. Profiles smaller than 1 μm diameter were not counted. Neuronal counting with NeuN staining included only those cells with a visible nucleus. Measurements were undertaken using the CAST 2.0 stereology system (Olympus Danmark A/S, Albertslund, Denmark) with an Olympus BX50 microscope and a one-chip colour CCD camera (TK-C138; JVC, Tokyo, Japan). Overall DRN and subnucleus cross-sectional area was also recorded. Cell size and staining intensity were measured using the MCID Elite v7.0 imaging system (Interfocus, Linton, UK) and a cooled CCD camera (SPOT RT Slider-2000; Diagnostic Instruments, USA) on a Nikon Eclipse E600 microscope. To determine size, cells were only measured where the whole cell outline was complete and well defined. Section thickness in the z-axis was measured using a microcator. The use of pre-cut thin frozen sections precluded the use of empirical or dissector stereological methods, so two-dimensional counts of PH8 profiles were corrected for split cells using the Abercrombie (1946) method on a subnucleus-by-level basis for each subject. Abercrombie correction was not used for NeuN, given the small size of cell nuclei relative to section thickness. See Supplementary Methods for additional details of cell counting protocols.

### 2.4 Regional and cellular in situ hybridization for 5-HT_1A_R mRNA

ISH followed the method of Burnet et al. (1995a), detailed in Supplementary Methods, and used two ^35^S-labelled oligonucleotides. Hybridized sections were apposed to X-ray film (Biomax MR; Kodak, USA) for three weeks with ^14^C microscales. For cellular analysis, sections were dipped in photographic emulsion (LM-1; Amersham Pharmacia Biotech, UK) mixed 2:1 with 2% glycerol, dried overnight, stored at 4 °C for seven weeks, developed in diluted Phenisol (Ilford, USA), cleared in 2% acetic acid, fixed in 30% sodium thiosulphate, and lightly stained with cresyl violet.

Densitometric analysis of autoradiographic films was carried out using the MCID system and a cooled CCD camera (CoolSNAP_cf_; Photometrics, USA) with a light box, and shading correction. The DRN was delineated and the integrated density measured with greyscale values converted into nCi/g tissue equivalents using a standard curve determined from the ^14^ C microscale. These values were corrected for local background. The cross-sectional area of DRN signal was measured. Emulsion-dipped sections were analysed in subnuclei of the DRN with the MCID microscope system, using a 20 × objective, delineating 5-HT_1A_R mRNA-positive cells under light-field conditions and measuring the corresponding optical density of overlying silver grains under dark-field illumination. These measures were corrected for local background.

### 2.5 Statistical analysis

All statistical analyses were carried out in SPSS 13.0 with a per-test type I error rate of 5%. Tests were carried out at the three DRN rostrocaudal levels, using the mean value for all sections at each level (1–3 sections per level). Results are given for all subnuclei combined; for subnucleus-specific findings (Levels 2 and 3) see Supplementary Material. Ideally a repeated-measures ANOVA would be used, however, this was not feasible, as many subjects did not have values for all levels. Therefore, the analysis was done ‘per level’, with planned comparisons of the control subjects versus the three other diagnostic groups using ANOVA. To analyse effects of suicide, subjects were divided into three groups: controls (none of whom died by suicide), patients who committed suicide, and patients who did not. Statistically significant correlations with demographic and confounding variables were included in an analysis of covariance (ANCOVA), with a non-parametric rank-transform ANCOVA where appropriate (see Supplementary Methods).

## 3 Results

### 3.1 Normative data

Fig. 1 shows the distribution of PH8-immunoreactive 5-HT neurons within the DRN and its subnuclei at the three rostrocaudal levels studied. Neuronal types were consistent with those found previously (Baker et al., 1990, 1991; Baumann et al., 2002), including large round neurons in the ventral subnucleus (DRv) (with some in the interfascicular subnucleus [DRif]), elongated fusiform cells in the DRif, large ovoid neurons in the dorsal subnucleus (DRd), and small ovoid and triangular neurons found in all subnuclei. Neuronal distributions were also broadly similar to prior reports (Baker et al., 1990, 1991; Baumann et al., 2002; Bielau et al., 2005; Underwood et al., 1999), except that the density of cells in the ventrolateral subnucleus (DRvl) appears to be much less in our study than found by Baker et al. (1990, 1991).

**Fig. 2:**
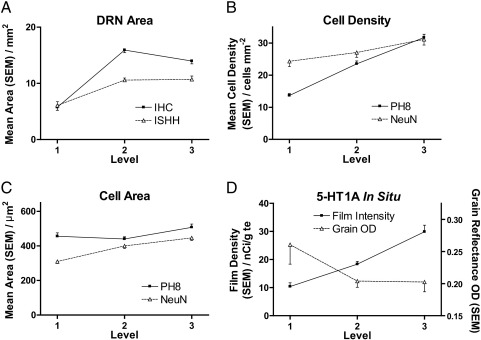
Rostrocaudal gradients across the human DRN. For definitions of the three anatomical levels, see text and Fig. 1. A: Cross-sectional DRN area, as measured by the extent of NeuN immunostaining (squares), and the extent of 5-HT_1A_R mRNA hybridization signal (triangles). B: Density of cells immunolabelled by NeuN (triangles) and PH8 (squares). C: Cross-sectional area of cells immunolabelled by NeuN (triangles) and PH8 (squares). D: Relative abundance of 5-HT_1A_R mRNA, as measured on film autoradiograms (left axis, squares) and per cell (right axis, triangles). Each panel shows the average for all subjects; similar profiles are seen for the control group alone (data not shown).

Across all subjects, DRN area, defined either by extent of NeuN and PH8 immunostaining or 5-HT_1A_R mRNA signal, increased from Level 1 to Levels 2 and 3 (Fig. 2A; all pairwise comparisons p < 0.05). The area of the DRN correlated with fresh brain weight across all subjects (Level 2 r = 0.48, p < 0.01; Level 3 r = 0.43, p = 0.05). For area of DRN subnuclei at each level, see Supplementary Table 1.

PH8 immunopositive cell density increased from Level 1 to Level 3 (Fig. 2B; all pairwise comparisons p < 0.01), while NeuN cell density showed a less marked increase (Fig. 2B; significant difference between Levels 1 and 3, p < 0.05), indicating that the proportion of serotonergic neurons increases rostrocaudally within the DRN. See Supplementary Tables 2 and 3 for PH8 and NeuN cell densities in each subnucleus at each level.

The mean size of NeuN-stained neurons increased from Level 1 to Level 3 (Fig. 2C; significant between Level 1 and the other levels, p < 0.01; Supplementary Table 5), but with no significant variation for PH8-immunopositive cells. For subnucleus-specific data on cell size, see Supplementary Tables 4 and 6.

The intensity of DRN 5-HT_1A_R ISH signal increased from Level 1 to Level 3 (Fig. 2D), paralleling the increasing proportion of cells that are PH8 positive (Fig. 2B), but the signal intensity per cell did not change between levels (Fig. 2D).

There were no correlations between study measures and age, pH, PMI, freezer storage time, gender, history of medication or substance misuse, or onset and duration of illness except where mentioned below.

### 3.2 Area of the DRN

**Fig. 3:**
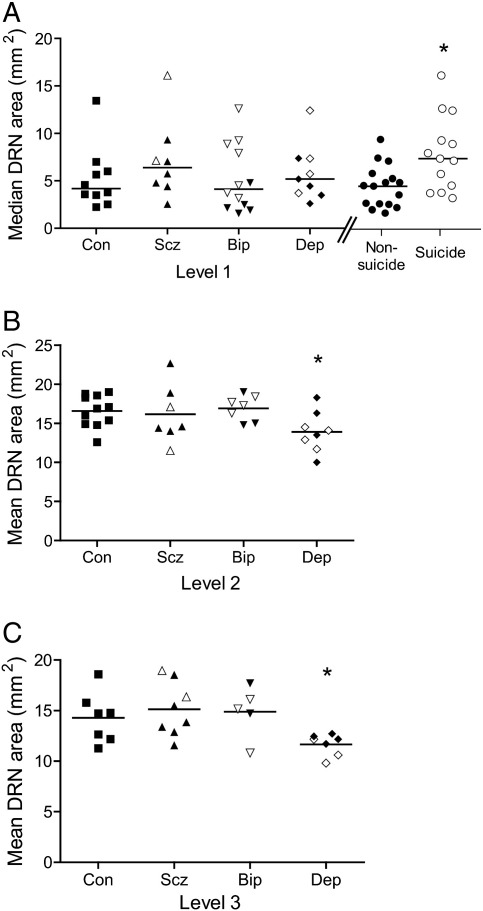
Cross-sectional area of the DRN, as delineated by immunostaining, at the three rostrocaudal levels, grouped by diagnosis. A: Level 1. B: Level 2. C: Level 3. At Level 1, patients are also separately grouped together by the presence or absence of suicide. Filled markers show non-suicides, and unfilled symbols show suicides. *p < 0.05.

Two differences in DRN area were seen between groups (Fig. 3). First, DRN area was reduced in subjects with MDD compared to controls at Level 2 (planned contrast, p = 0.05) and Level 3 (ANOVA p < 0.05; planned contrast, p < 0.05). The reduction was apparent in all subnuclei at this level (Supplementary Table 1). Second, at Level 1, DRN area was increased in patients committing suicide compared to other patients (Kruskal–Wallis [KW] ANOVA p < 0.05; Wilcoxon–Mann–Whitney [WMW] suic > non-suic p < 0.05); inspection of Fig. 3A indicates that this difference is seen across diagnostic groups). Including brain weight as a covariate in analyses eliminated the significance of the reduction of Level 2 DRN area in MDD (ANCOVA p = 0.53; planned contrast, p = 0.18) and Level 3 (ANCOVA p = 0.09; planned contrast p = 0.10) but the increased area in suicide survived (rank-transform ANCOVA p = 0.03; planned contrasts p < 0.05).

### 3.3 Neuronal density in the DRN

**Fig. 4:**
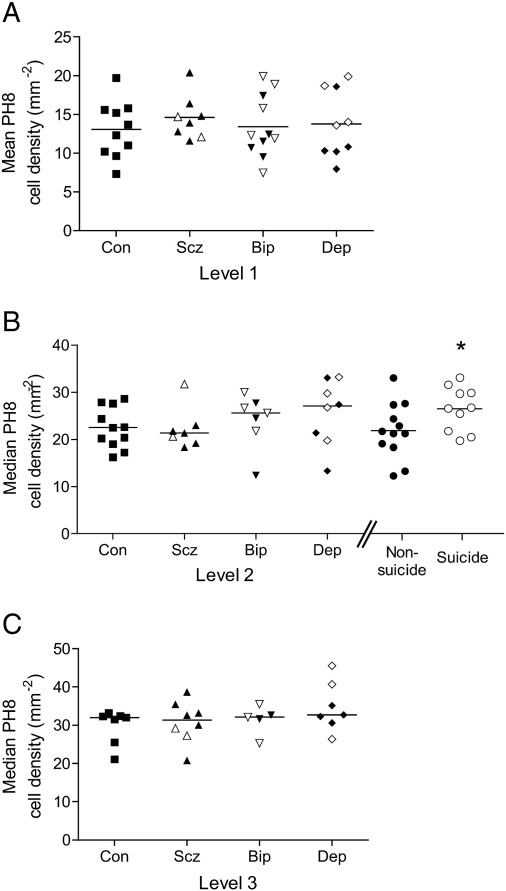
Density (cells/mm^2^) of PH8-immunopositive cells in the DRN at the three rostrocaudal levels, grouped by diagnosis. Black symbols show non-suicides, and white symbols show suicides. A: Level 1. B: Level 2. C: Level 3. At Level 2, patients are also separately grouped together by the presence or absence of suicide. *p < 0.05.

Abercrombie corrected PH8 immunopositive cell counts did not reveal any diagnostic differences (Fig. 4). At Level 2, PH8 cell density was increased in patients committing suicide versus non-suicides (planned contrast, p < 0.05; Fig. 4B). PH8 cell density at Level 2 correlated with post-mortem interval (r_s_ = − 0.38, p < 0.05) and the comparison became a non-significant trend when this was included as a covariate (p = 0.055), but became significant for suicides versus controls (planned contrast, p < 0.05). NeuN cell density significantly correlated with PH8 cell density (r = 0.38, p < 0.0005) but did not differ by diagnosis at any level (Table 2). NeuN cell density inversely correlated with freezer storage time (Level 1 r = − 0.38 p < 0.05; Level 2 r = − 0.38 p < 0.05; Level 3 r = − 0.45 p < 0.05), pH (Level 1 r = 0.51 p < 0.01; Level 2 r = 0.39 p < 0.05), and post-mortem interval (Level 3 r = − 0.49 p < 0.05). Inclusion of these factors as covariates did not affect the results. See Supplementary Tables 2 and 3.

### 3.4 Neuronal soma size in DRN

**Fig. 5:**
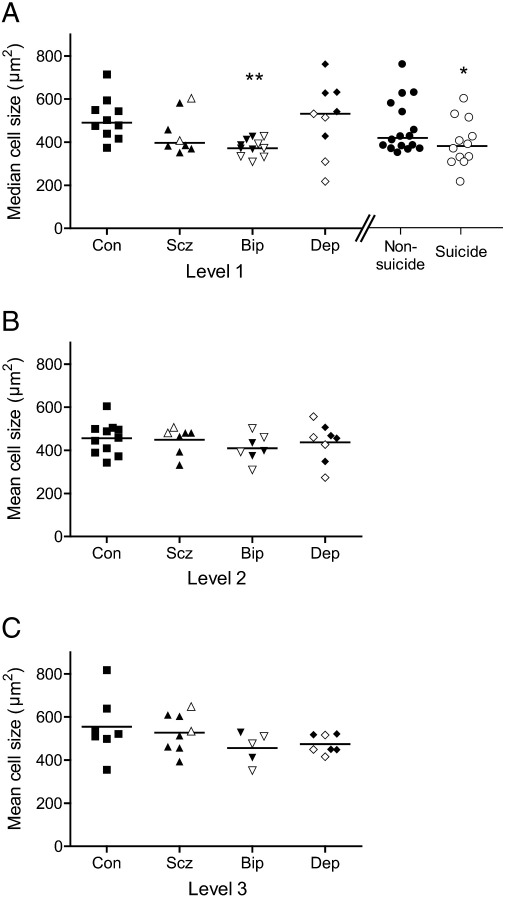
Cross-sectional area (μm^2^) of the soma of PH8-immunopositive cells in the DRN at the three rostrocaudal levels, grouped by diagnosis. A: Level 1. B: Level 2. C: Level 3. At Level 1, patients are also separately grouped together by the presence or absence of suicide. Black symbols show non-suicides, and white symbols show suicides. **p < 0.01.

At Level 1 the area of PH8 neurons was less in bipolar disorder subjects compared to controls (KW ANOVA p < 0.05; WMW bip < con p < 0.01; Fig. 5) and decreased in suicides versus controls (KW ANOVA p < 0.05; WMW suic < con p < 0.05; Fig. 5). Freezer storage time correlated with PH8 cell area at Level 1 (r_s_ = − 0.33, p < 0.05) and Level 2 (r = − 0.37, p < 0.05); with freezer storage time as covariate the reductions in cell size at Level 1 seen in bipolar disorder and suicide groups remained significant. There were no differences in NeuN-immunoreactive cell size. See Supplementary Tables 4–6.

### 3.5 5-HT_1A_R mRNA abundance in DRN and DRN neurons

**Fig. 6:**
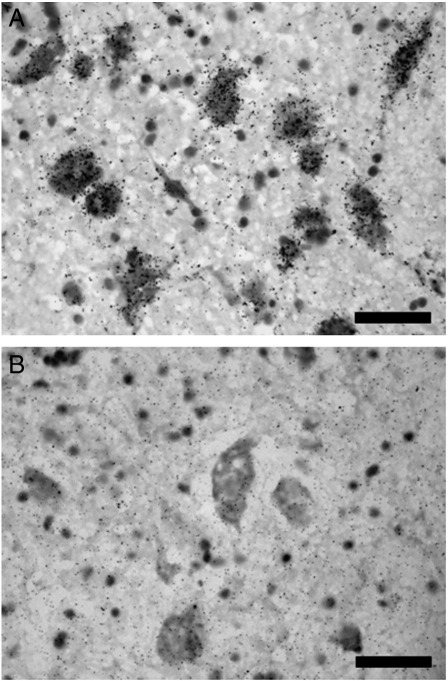
A: Cellular localization of 5-HT_1A_R mRNA in the human DRN. B: Sense strand control, showing low level of background labelling. Sections counterstained with cresyl violet. Scale bar = 50 μm.

The spatial extent of 5-HT_1A_R mRNA signal correlated with DRN area delineated by immunostaining (r = 0.68, p < 0.001), and replicated the significant reduction in DRN area at Level 2 in subjects with MDD (planned contrast, p < 0.05) but not at Level 3 (data not shown). Emulsion-dipped sections revealed that hybridization signal for 5-HT_1A_R mRNA was concentrated over large cells, putatively 5-HT neurons (Austin et al., 1994; Fig. 6), with no clear differences in cellular grain density between DRN subnuclei (Supplementary Table 7).

DRN 5-HT_1A_R mRNA film signal correlated inversely with post-mortem interval (Level 2, r = − 0.35 p < 0.05; Level 3, r = − 0.41 p < 0.05), and positively with pH (Level 3, r = 0.48 p < 0.01). With these factors as covariates, DRN 5-HT_1A_R mRNA signal did not differ between diagnostic groups nor by suicide status (Table 3). The expression of 5-HT_1A_R mRNA per cell within the DRN correlated with the overall DRN hybridization signal intensity (r = 0.37, p < 0.05); it also related inversely with freezer storage time (Level 1 r_s_ = − 0.49 p < 0.05; Level 2 r_s_ = − 0.41 p < 0.05; Level 3 r_s_ = − 0.38 p < 0.05) and post-mortem interval (Level 2 r_s_ = − 0.42 p < 0.05; Level 3 r_s_ = − 0.44 p < 0.05), and positively with pH (Level 3 r_s_ = 0.56 p < 0.01). The grain density of dipped sections differed in the DRN taken as a whole at Level 3 (WMW bip < con p < 0.05) but became non-significant when freezer storage time, PMI, and pH were added as covariates (Supplementary Table 7 and Supplementary Figs. 5–7).

## 4 Discussion

A role for 5-HT in mood disorders and in suicide has long been suspected, and neuropathological studies of the DRN have been one manifestation of this interest. However, to date, the studies have had several limitations, most notably their small sample size, and also the fact that in most studies (except two studies of late life depression: Hendricksen et al., 2004; Syed et al., 2005), virtually all mood disorder subjects died by suicide, meaning that the possibility of differential effects of diagnosis and mode of death could not be disentangled.

This study attempted to overcome some of these limitations by using a larger sample, and by including subjects with mood disorders who did not die by suicide, and suicide victims who had schizophrenia. In this way we hoped to parse the effects of diagnosis from those of suicide. And, we used three complementary indices to assess DRN status: NeuN as a marker of all neurons; PH8 immunostaining for 5-HT neurons, and 5-HT_1A_R mRNA in situ hybridization as another means to identify 5-HT neurons and their expression of this key inhibitory autoreceptor. Our main findings are that: 1) suicide is associated with an increased DRN area and with an increased density and decreased size of serotonergic neurons; 2) in MDD, DRN area is decreased, with no differences in cell density or size, and 3) in bipolar disorder, serotonergic neuron size is decreased. These findings were limited, in terms of anatomical extent or robustness to confounding factors. We found no alterations in schizophrenia, and no diagnosis- or suicide-associated differences in 5-HT_1A_R mRNA expression.

### 4.1 Differences in DRN area and cell composition in suicide and mood disorders

Subjects committing suicide showed an increased area of the rostral pole (Level 1) of the DRN (with decreased serotonergic cell size and no change in cell density) and an increased TPH-immunoreactive (i.e. serotonergic) cell density more caudally (Level 2) within the rostral DRN. The latter agrees with a finding by Underwood et al. (1999) in suicide victims most of whom had MDD; another group have reported a similar observation in suicide victims with either unipolar or bipolar disorder which was limited to the ventrolateral subnucleus (Baumann et al., 2002; Bielau et al., 2005).

Note that we did not find a corresponding increase in total neuronal density as measured by NeuN, and that the definition of a cell type as ‘serotonergic’ is relative and not absolute, since it is affected by the abundance of the marker being used. Thus, it is noteworthy that suicide victims have increased DRN levels of TPH immunoreactivity (Boldrini et al., 2005), and of TPH2 mRNA (Bach-Mizrachi et al., 2006), including a greater abundance per neuron (Bach-Mizrachi et al., 2008). A parsimonious interpretation is therefore that suicide is associated with increased TPH which leads to a greater number of neurons being labelled as serotonergic, rather than more serotonergic neurons *per se*. In turn, it has been speculated that the up-regulation of TPH reflects a homeostatic response to impaired serotonin transmission in suicide (Bach-Mizrachi et al., 2006, 2008; Boldrini et al., 2005), especially when alterations in 5-HT_1A_R and 5-HT transporter expression are considered as well (Arango et al., 2001; Boldrini et al., 2008). However, these conclusions remain provisional, since another group found no difference in TPH immunoreactivity in suicide victims (Bonkale et al., 2004). Likewise, our finding of a decreased size of serotonergic neurons in rostral DRN in suicide victims may suggest a decreased axodendritic arborisation or activity, but requires replication since it was not observed by Underwood et al. (1999) or Arango et al. (2001).

Positive findings related to diagnosis were modest, and their interpretation hampered by the lack, noted earlier, of comparable studies in which diagnostic effects were studied independent of suicide. In MDD, we found the cross-sectional area of the DRN was decreased, without changes in cell size or density. This suggests a reduction in the neuropil, which is mainly comprised of glia, dendrites, axons, and vascular elements. In the cerebral cortex, several studies have reported glial decreases in MDD (e.g. Cotter et al., 2001; Gittins and Harrison, 2011; Öngür et al., 1998; Rajkowska et al., 1999), and a morphometric study of glia in the DRN would be of interest. Unchanged serotonergic neuronal density and size in DRN has also been reported in the two existing studies of elderly subjects with unipolar depression (Hendricksen et al., 2004; Syed et al., 2005). In bipolar disorder, we found serotonergic neurons in the rostral DRN to be decreased in somal area; this is reminiscent of studies showing that the size of some cortical neuron populations is reduced in the disorder (see Harrison, 2002). In schizophrenia, our negative findings for neuronal density and size replicated the one previous study (Craven et al., 2005), and we also found DRN area to be unaltered. As such, any serotonergic involvement in schizophrenia pathophysiology does not appear to include morphometric changes in the DRN.

### 4.2 DRN 5-HT_1A_R mRNA expression in mood disorders, suicide, and schizophrenia

The extent of 5-HT_1A_R mRNA distribution was decreased in MDD, consistent with the reduced DRN area measured immunohistochemically, and in agreement with a reduced extent of 5-HT_1A_R binding seen in an earlier study (Arango et al., 2001; Boldrini et al., 2008). However, we found no differences in 5-HT_1A_R mRNA abundance within the DRN (either overall or per neuron), in any diagnostic group, nor in suicide victims, suggesting that the expression of this key inhibitory autoreceptor is not altered in these conditions. To our knowledge this is the first study of this kind, although Anisman et al. (2008) state (without presenting the data) that they also found no alteration in subjects with depression or following suicide, and Goswami et al. (2010) found no alterations in 5-HT_1A_R mRNA from laser-capture microdissected DRN serotonergic neurons in MDD. In contrast, 5-HT_1A_R mRNA is decreased in the frontal cortex and hippocampus of MDD subjects in the present brain series (Lopez-Figueroa et al., 2004). The lack of a parallel (or reciprocal) change in the DRN is reminiscent of other situations, in which expression of the 5-HT_1A_R autoreceptor is unaltered despite changes in the post-synaptic 5-HT_1A_R population (e.g. after electroconvulsive shock; Burnet et al., 1995b). Studies of DRN 5-HT_1A_R binding in depressed suicides have given variable results, but with some evidence for increased binding rostrally and decreased binding caudally (Arango et al., 2001; Boldrini et al., 2008; Stockmeier et al., 1998). Altered 5-HT_1A_R binding with no change in encoding mRNA has been observed previously, and may reflect a prominent post-transcriptional component to receptor regulation (Burnet et al., 1996).

### 4.3 Limitations

Despite being the largest study of its kind, our work has several limitations. Most importantly the extent of the tissue and the characteristics of the sections. The whole DRN was not sampled; thus, although most neurons, of the DRN are found in the rostral subdivisions sampled here (Baker et al., 1990), estimates of DRN volume and cell number are not possible. And, since not all subjects had tissue from all three anatomical levels of the DRN, the rostrocaudal length of the DRN and its subnuclei could not be determined, and anatomical level could not be included in a repeated-measures ANOVA. Finally, given the thickness of the sections (14 μm before processing, ~ 6 μm once stained) three-dimensional counting procedures were not possible, requiring two-dimensional ‘non-stereological’ methods, which have inherent limitations. For example, although we used the Abercrombie correction for PH8 cell counts, this is imperfect and does not control for ‘lost caps’ (Hedreen, 1998), and the corrected cell densities will remain somewhat inaccurate due to the large and heterogeneous size of DRN neurons (Guillery and Herrup, 1997). However, for comparative studies between groups, this should not bias the profile counts unless there are corresponding and marked differences in cell size (which did not prove to be the case; if anything, the decreased cell size in suicide will have mitigated against finding an increased cell density).

A second weakness is the limited information available regarding medication and substance misuse. Quantitative estimates of lifetime exposure were available for antipsychotics, but only dichotomous (yes/no) information was available about antidepressants and mood stabilizers. It is therefore possible that some of our findings, both positive and negative, may have been confounded by these drugs. Such confounding is most plausible for the 5-HT_1A_R mRNA data, since expression of this receptor is known to be altered by various depression treatments (Burnet et al., 1994, 1995b, 1999; Chen et al., 1995). Similarly, PH8 immunostaining could be affected by antidepressant-induced alterations in abundance of TPH isoforms (Bielau et al., 2005; Shishkina et al., 2007; Yang et al., 2010). There is an association between smoking, suicide, and low serotonergic markers (Malone et al., 2003) but we did not have smoking data for these subjects to examine the relationship. There is also an association between alcoholism and decreased serotonin transmission (Fahlke et al., 2011), although there is no evidence of serotonergic neuron loss or change in cell size in the DRN in alcoholism (Baker et al., 1996; Underwood et al., 2007), and we did not find any effect of substance, including alcohol, misuse.

Thirdly, our data highlight the potential sensitivity of morphological and immunocytochemical indices to peri- and post-mortem variables, including pH, post-mortem interval and freezer storage time (Harrison et al., 1995). Such factors are well appreciated in molecular studies, but not always considered in morphometric and immunohistochemical ones.

### 4.4 Conclusions

We found evidence for modest alterations in DRN morphology in suicide, MDD, and bipolar disorder. These data add to the evidence that the serotonergic involvement in mood disorder and suicide includes a presynaptic and ‘neuropathological’ component. Our finding of differential effects of MDD and suicide provides one explanation for why previous studies (which have confounded the two) have had inconsistent findings. Together with the apparent anatomical heterogeneity of changes, the findings indicate that further progress will require larger studies, of the complete DRN, from subjects who are well characterised clinically and demographically, and which also measure and take account of a range of confounding variables.

## Role of funding source

P.R.M. was funded by a Wellcome Trust Oxford Neurosciences M.Sc./D.Phil. studentship. The work was supported by a Medical Research Council ‘Neurobiology of Mood Disorders’ Co-Operative Group Award, and a Stanley Medical Research Institute Centre award to P.J.H. The funders had no role in study design; in the collection, analysis and interpretation of data; in the writing of the report; and in the decision to submit the paper for publication.

## Conflict of interest

Neither author declares any conflict of interest.

## Figures and Tables

**Table 1:** Demographics of subjects studied.

	Control	Schizophrenia^a^	Bipolar disorder^b^	Major depression
Subjects	13	10	13	14
Age (years)	48.7 (10.8)	43.2 (13.0)	42.4 (12.2)	46.6 (9.7)
Sex (M, F)	8, 5	8, 2	8, 5	8, 6
Brain pH	6.25 (0.24)	6.12 (0.29)	6.17 (0.25)	6.19 (0.22)
Fresh brain weight (g)	1521 (167)	1516 (98)	1444 (180)	1439 (133)
Freezer storage (months)^c^	92.7 (8.1)	102.1 (5.6)	102.8 (4.7)	95.6 (10.0)
Suicides	0	3	8	6
Onset of illness (years)	–	22.9 (8.0)	21.4 (8.9)	34.4 (13.7)
Duration of illness (years)	–	21.0 (10.7)	21.6 (9.7)	12.3 (11.4)
Lifetime antipsychotic dose^d^	0	47,800 (54,788)	23,492 (6868)	0
Antipsychotics ever	0	10	11	0
Antidepressants ever	0	3	6	9
Mood stabilisers ever	0	0	8	2
Current substance abuse or dependence	0	2	4	3

Values are mean (S.D.) where appropriate.

a Subtypes: paranoid (n = 2), undifferentiated (n = 8).

b 10 subjects had psychotic features.

c Differs between groups (ANOVA p = 0.003; controls < schizophrenia (p = 0.005) and bipolar disorder (p = 0.001).

d Fluphenazine equivalents (g).

**Table 2:** Density of NeuN-labelled neurons in the DRN.

	Controls		Schizophrenia		Bipolar disorder		Major depression		Suicides		Non-suicides^a^	
Level	Mean (SEM)	N	Mean (SEM)	N	Mean (SEM)	N	Mean (SEM)	N	Mean (SEM)	N	Mean (SEM)	N
1	26.1 (1.8)	8	20.2 (3.8)	8	22.0 (2.5)	11	30.5 (4.2)	7	27.3 (3.1)	12	20.7 (2.5)	14
2	28.2 (1.8)	10	21.2 (3.6)	5	26.4 (3.6)	6	30.1 (3.4)	7	26.7 (2.5)	8	26.1 (3.3)	10
3	31.7 (3.1)	7	29.5 (3.1)	6	27.8 (4.5)	5	35.7 (4.0)	5	32.1 (2.8)	7	30.0 (3.4)	9

Values are cells per mm^2^. There are no significant differences between groups.

a Excludes normal controls.

**Table 3:** 5-HT_1A_R mRNA signal in the DRN measured with film autoradiography.

	Controls		Schizophrenia		Bipolar disorder		Major depression		Suicides		Non-suicides^a^	
Level	Mean (SEM)	N	Mean (SEM)	N	Mean (SEM)	N	Mean (SEM)	N	Mean (SEM)	N	Mean (SEM)	N
1	9.9 (1.1)	4	7.6 (2.2)	4	20.8 (5.9)	2	9.3 (1.4)	7	13.2 (2.7)	7	7.4 (1.2)	6
2	18.9 (1.4)	11	15.9 (2.6)	8	17.1 (2.4)	7	20.6 (1.9)	10	20.2 (1.9)	13	15.9 (1.6)	12
3	32.0 (2.3)	8	22.9 (5.3)	8	25.9 (5.3)	6	38.7 (4.2)	7	31.1 (4.3)	10	27.1 (4.6)	11

Values are nCi/g tissue equivalents. There are no significant differences between groups.

a Excludes normal controls.
