# Supplementary material for: A morphometric, immunohistochemical, and in situ hybridization study of the dorsal raphe nucleus in major depression, bipolar disorder, schizophrenia, and suicide
Source: J Affect Disord. 2012 Mar;137(1-3):125–34. doi: 10.1016/j.jad.2011.10.043 (PMC3314923; doi:10.1016/j.jad.2011.10.043)
Supplement: Supplementary file 1 — Supplementary materials [file mmc1.doc]

**Supplementary Material for:**

**A morphometric and immunohistochemical study of the dorsal raphe nucleus in major depression, bipolar disorder, schizophrenia, and suicide**

Paul R. Matthews, Paul J. Harrison

**Comprising:**

**Supplementary Methods**

**Supplementary Results**

**Suppementary Tables**

**Supplementary Figures**

**Supplementary Methods**

*Immunohistochemistry for NeuN and PH8*

Sections were fixed for 5mins in 4% paraformaldehyde, dehydrated through graded alcohols, pre-treated in 3% hydrogen peroxide in methanol for 30mins, rehydrated, incubated for 30mins at room temperature (RT) in 10% normal horse serum (NHS) in PBS-T (phosphate buffered saline + 0.4% Triton X-100 detergent), washed in PBS (3x10mins), incubated in the primary antibody (1% NHS, PBS-T) in a humidified chamber, washed, incubated in the secondary biotinylated antibody (1% NHS, PBS-T; 1 hr RT), washed, incubated in 1% avidin-biotin peroxidase complex (1% NHS, PBS-T; 1hr RT), washed and finally the bound horseradish peroxidase was revealed using 3,3’-diaminobenzidine (DAB; 0.025% in PBS + 0.06% hydrogen peroxide; 5mins) as chromogen. Sections were washed, dehydrated, cleared in xylene and then mounted and coverslipped.

*Cell counting and morphometry*

Level 1 is the rostral pole (DRr) of the DRN, at the level of the oculomotor nucleus; Level 2 lies between the caudal pole of the oculomotor nucleus and the rostral end of the trochlear nucleus and contains the dorsal (DRd), ventral (DRv) and interfascicular (DRif) subnuclei; at Level 3 the DRN is at its greatest width, with the ventrolateral subnucleus (DRvl) present in addition to the DRd, DRv and DRif.

In the horizontal plane we used an unbiased counting frame covering 80% of the visible part of the section using systematic random sampling on a per subnucleus basis (cell density frame 27,383 μm^2^; cell size frame 60,621 μm^2^; dipped section frame 15,155 μm^2^). PH8-positive cell counts were obtained with a 40x objective, randomly sampling 100-200 frames per subnucleus systematically, counting approximately 100 cells per subnucleus. NeuN-positive cells were resolved with a 100x oil immersion lens to discriminate the nucleus, counting 200-400 frames per subnucleus, and thus approximately 50 cells per subnucleus. Mean cross-sectional somal area was used as the measure of cell size, obtained with a 40x objective, with fifty cells (PH8) or thirty cells (NeuN) per subnucleus per subject sampled to estimate average cell size. These frame and profile numbers were determined from pilot studies to provide repeated counts within 5%. In the vertical plane profiles were counted through the entire thickness due to the use of very thin sections.

*Regional and cellular in situ hybridisation for 5-HT_1A_R mRNA*

Sections were pre-treated by fixing in 4% paraformaldehyde for 5mins, rinsing twice in PBS, acetylated in 0.25% acetic anhydride (in 0.1M triethanolamine; pH 8.0) for 10 mins, dehydrated and delipidated in a graded series of ethanol washes and chloroform. The slides were partially rehydrated through 100% and 95% ethanol for 1min, dried and then stored at -20°C. An equimolar mixture of two antisense oligonucleotides against sequences within the 5-HT_1A_R transcript which are homologous in human and rat (ctttggagttgcccactcggtgcacctcgatcacctcca; Cgagaggtgatcacttggtagctgacggtcacgtcggagat) were 3'-labelled with α-[^35^S]-dATP. Sections were covered in 200µl of hybridisation buffer (600 mmol sodium chloride/60 mmol sodium citrate, 50% deionized formamide, 10% dextran sulphate, 5x Denhardt’s solution, 200 mg/ml salmon testes DNA, 100 mg/ml poly[A], 25 mM sodium phosphate pH 7, 1 mM sodium pyrophosphate, 120 mg/ml heparin) containing 5000 counts/µl of labelled probe and 50mM dithiothreitol and coverslipped. Incubations were carried out for 18hrs at 35°C in a humidifying chamber. Post-hybridisation washes were in 150mmol sodium chloride/15mmol sodium citrate at 59°C (3x20mins) and RT (2x60mins), followed by a rinse in double distilled water.

Negative ISH controls were carried out using sense probes, and displacement with twenty-fold excess unlabelled probe; positive controls included sections of the rat DRN.

*Statistical analysis*

Samples failing Shapiro-Wilk or Levene’s tests were analysed with a Kruskal-Wallis (KW) non-parametric ANOVA and comparisons between the diagnostic and control groups carried out using a Wilcoxon-Mann-Whitney (WMW) two-sample statistic.

To investigate potentially confounding demographic and peri-mortem variables, Pearson (and Spearman where appropriate) correlations were carried out at α = .05 and checked visually. Significant correlations led to the variable being included in an analysis of covariance (ANCOVA). The main factors examined were age, pH, PMI, and freezer storage time (the latter was confounded with diagnosis, see Table 2). For variables failing tests for normality or homogeneity of variance, the rank-transform ANCOVA represents a suitable non-parametric statistic for including covariates (Conover and Iman, 1982; Olejnik and Algina, 1984) and was used here. Exploratory correlations and comparisons were carried out using other variables, including gender, brain weight, history of medication or substance misuse, and onset and duration of illness.

**Supplementary Results**

As with other brain regions the brainstem shows shrinkage when processed (Quester and Schroder, 1997) and differential shrinkage of section thickness in subjects with depression has been reported (Stockmeier et al., 2004). Therefore section thickness was measured at the level of the oculomotor nucleus (Level 2). It did not differ between diagnostic groups, nor did it correlate with any demographic variables.

Variability in PH8 staining intensity was found in a prior study to correlate with neuronal density, likely reflecting a difficulty in detecting poorly stained cells (Craven et al., 2005). Here, average per cell staining intensity was measured (at Level 2); it did not differ between groups, nor correlate with demographic variables.

To assess reliability, five brains were selected at random and cells recounted for both NeuN and PH8 morphometric measures. Intra-class correlation coefficients, calculated on a per subnucleus basis, were all > 0.95.

We did not include estimates of neuronal numbers in main results because we present both neuronal density and DRN area so reporting all three would be redundant and statistical testing would not be independent. However, we present this data in Supplementary Tables 8-9 where the only statistically significant findings are an increase in cell number (both PH8 and NeuN-positive) at the most rostral level (Level 1) in suicide which corresponds with the finding that the area of the DRN is increased at this level and suggests that this larger size is secondary to increased cell number.

Conover, W. J., Iman, R. L. 1982. Analysis of covariance using the rank transformation. Biometrics 38, 715-24.

Craven, R. M., Priddle, T. H., Cooper, S. J., Crow, T. J., Esiri, M. M. 2005. The dorsal raphe nucleus in schizophrenia: a post mortem study of 5-hydroxytryptamine neurones. Neuropathol. Appl. Neurobiol. 31, 258-69.

Olejnik, S. F., Algina, J. 1984. Parametric ANCOVA and the rank transform ANCOVA when the data are conditionally non-normal and heteroscedastic. J. Educ. Stat. 9, 129-49.

Quester, R., Schroder, R. 1997. The shrinkage of the human brain stem during formalin fixation and embedding in paraffin. J. Neurosci. Meth. 75, 81-9.

Stockmeier, C. A., Mahajan, G. J., Konick, L. C., Overholser, J. C., Jurjus, G. J., Meltzer, H. Y., Uylings, H. B., Friedman, L., Rajkowska, G. 2004. Cellular changes in the postmortem hippocampus in major depression. Biol. Psychiatry 56, 640-50.

**Supplementary Tables**

**Supplementary Table 1.** DRN Subnucleus Area (mm^2^) As Delimited by PH8 Immunohistochemistry

|  | | Con | | Scz | | Bip | | Dep | | Suic | | Non-Suic | |
| --- | --- | --- | --- | --- | --- | --- | --- | --- | --- | --- | --- | --- | --- |
| Level | Nucleus | Mean (SEM) | N | Mean (SEM) | N | Mean (SEM) | N | Mean (SEM) | N | Mean (SEM) | N | Mean (SEM) | N |
| 2 | DRif | 2.02 (.145) | 11 | 2.09 (.174) | 6 | 2.15 (.167) | 7 | 1.91 (.177) | 8 | 1.89 (.154) | 9 | 2.15 (.124) | 12 |
|  | DRv | 3.09 (.184) | 11 | 2.75 (.227) | 7 | 3.17 (.193) | 7 | 2.78 (.183) | 8 | 2.92 (.151) | 10 | 2.87 (.181) | 12 |
|  | DRd | 11.5 (.496) | 11 | 11.7 (1.05) | 7 | 11.6 (.620) | 7 | 9.21 (.756)^a^ | 8 | 10.5 (.611) | 10 | 10.9 (.826) | 12 |
| 3 | DRif | 1.45 (.222) | 8 | 2.07 (.234) | 8 | 1.56 (.136) | 5 | 1.06 (.151) | 6 | 1.50 (.246) | 8 | 1.71 (.192) | 11 |
|  | DRv | 2.91 (.221) | 7 | 2.80 (.208) | 8 | 2.90 (.259) | 5 | 2.19 (.142)^b^ | 7 | 2.44 (.181) | 8 | 2.73 (.180) | 12 |
|  | DRvl | 2.26 (.138) | 8 | 1.80 (.200) | 8 | 2.15 (.263) | 5 | 1.99 (.130) | 6 | 1.95 (.156) | 8 | 1.96 (.159) | 12 |
|  | DRd | 7.79 (.650) | 7 | 8.46 (.695) | 8 | 8.28 (.638) | 5 | 6.54 (.252) | 7 | 8.09 (.709) | 8 | 7.51 (.425) | 12 |
|  |  |  |  |  |  |  |  |  |  |  |  |  |  |

1. WMW dep<con p<.05 (Fig S1).
2. Planned contrast dep<con p<.05 (Fig S2).

**Supplementary Table 2.** Density of PH8-immunopositive Cells (mm^-2^) by Subnucleus

|  | | Con | | Scz | | Bip | | Dep | | Suic | | Non-Suic | |
| --- | --- | --- | --- | --- | --- | --- | --- | --- | --- | --- | --- | --- | --- |
| Level | Nucleus | Mean (SEM) | N | Mean (SEM) | N | Mean (SEM) | N | Mean (SEM) | N | Mean (SEM) | N | Mean (SEM) | N |
| 2 | DRif | 35.2 (3.37) | 11 | 22.7 (5.60) | 6 | 36.0 (6.02) | 7 | 31.2 (4.53) | 8 | 37.2 (3.11) | 9 | 25.3 (4.59) | 12 |
|  | DRv | 36.6 (2.58) | 11 | 36.3 (3.12) | 7 | 40.5 (3.86) | 7 | 38.6 (3.92) | 8 | 41.1 (.305) | 10 | 36.3 (2.74) | 12 |
|  | DRd | 16.4 (.86) | 11 | 19.1 (1.33) | 7 | 17.7 (1.32) | 7 | 20.6 (2.31)^a^ | 8 | 21.2 (1.43)^b^ | 10 | 17.5 (1.29) | 12 |
| 3 | DRif | 39.6 (3.07) | 8 | 37.8 (4.37) | 8 | 47.0 (2.37) | 5 | 50.1 (4.35) | 6 | 43.3 (4.14) | 8 | 44.7 (3.51) | 11 |
|  | DRv | 32.4 (2.25) | 7 | 36.0 (3.25) | 8 | 34.3 (3.94) | 5 | 40.4 (5.24) | 7 | 39.3 (5.09) | 8 | 35.6 (2.25) | 12 |
|  | DRvl | 46.4 (2.10) | 8 | 49.0 (2.92) | 8 | 52.5 (3.24) | 5 | 52.3 (2.90) | 6 | 53.5 (3.01) | 7 | 49.4 (2.02) | 12 |
|  | DRd | 22.4 (1.28) | 7 | 24.4 (1.90) | 8 | 23.0 (1.60) | 5 | 26.4 (1.92) | 7 | 24.8 (2.09) | 8 | 24.7 (1.22) | 12 |

a. With PMI as covariate: rank-transform ANCOVA planned contrast dep>con p<.05.

b. KW ANOVA p<.01; WMW suic>non-suic p<.05, suic>con p<.01 (Fig S3). With PMI as covariate: rank-transform ANCOVA p<.01; planned contrasts suic>non-suic p<.05, suic>con p<.01. This effect appears to hold across suicides in all diagnostic groups.

**Supplementary Table 3.** Density of NeuN-positive Cells (mm^-2^) by Subnucleus

|  | | Con | | Scz | | Bip | | Dep | | Suic | | Non-Suic | |
| --- | --- | --- | --- | --- | --- | --- | --- | --- | --- | --- | --- | --- | --- |
| Level | Nucleus | Mean (SEM) | N | Mean (SEM) | N | Mean (SEM) | N | Mean (SEM) | N | Mean (SEM) | N | Mean (SEM) | N |
| 2 | DRif | 32.0 (3.60) | 10 | 14.6 (4.34)^a^ | 5 | 26.3 (4.86) | 6 | 31.6 (5.08) | 7 | 28.5 (2.91) | 8 | 22.4 (5.15) | 10 |
|  | DRv | 34.9 (2.34) | 10 | 31.6 (4.37) | 6 | 32.1 (3.83) | 6 | 33.1 (4.79) | 7 | 31.6 (3.11) | 9 | 32.9 (3.77) | 10 |
|  | DRd | 25.4 (1.99) | 10 | 21.2 (3.57) | 6 | 24.9 (3.90) | 6 | 28.6 (3.85) | 7 | 25.2 (2.52) | 9 | 25.0 (3.63) | 10 |
| 3 | DRif | 37.1 (4.37) | 8 | 27.8 (1.23) | 6 | 33.1 (5.78) | 5 | 45.8 (2.20) | 5 | 37.2 (3.44) | 8 | 33.4 (4.03) | 9 |
|  | DRv | 32.3 (4.64) | 7 | 33.7 (5.02) | 6 | 28.4 (5.50) | 5 | 34.7 (5.02) | 6 | 35.3 (5.32) | 7 | 30.5 (3.18) | 10 |
|  | DRvl | 42.9 (4.75) | 8 | 43.1 (5.48) | 6 | 37.6 (6.01) | 5 | 41.9 (5.48) | 6 | 44.6 (4.12) | 7 | 38.6 (4.75) | 10 |
|  | DRd | 27.6 (2.52) | 7 | 25.9 (3.11) | 6 | 23.7 (3.85) | 5 | 26.4 (4.77) | 6 | 27.4 (3.15) | 7 | 24.0 (3.03) | 10 |

1. a. Planned contrast scz<con p<.05 (Fig S4). With freezer storage time, pH, and PMI as covariates: planned contrast scz<con p=.06. With pH and PMI as covariates: planned contrast scz<con p<.05.
2. **Supplementary Table 4.** Cross-sectional Area of PH8-positive Cells (μm^2^) by Subnucleus

|  | | Con | | Scz | | Bip | | Dep | | Suic | | Non-Suic | |
| --- | --- | --- | --- | --- | --- | --- | --- | --- | --- | --- | --- | --- | --- |
| Level | Nucleus | Mean (SEM) | N | Mean (SEM) | N | Mean (SEM) | N | Mean (SEM) | N | Mean (SEM) | N | Mean (SEM) | N |
| 2 | DRif | 428 (28.0) | 11 | 419 (29.4) | 6 | 407 (25.6) | 7 | 402 (21.8) | 8 | 404 (27.0) | 9 | 412 (14.6) | 12 |
|  | DRv | 444 (31.7) | 11 | 469 (27.7) | 7 | 391 (24.0) | 7 | 423 (38.0) | 8 | 421 (35.8) | 10 | 433 (17.8) | 12 |
|  | DRd | 468 (20.7) | 11 | 439 (24.6) | 7 | 418 (30.4) | 7 | 446 (37.5) | 8 | 448 (29.8) | 10 | 424 (21.8) | 12 |
| 3 | DRif | 478 (47.5) | 8 | 482 (43.2) | 8 | 364 (40.7) | 5 | 397 (31.6) | 6 | 438 (40.3) | 8 | 415 (33.4) | 11 |
|  | DRv | 534 (58.6) | 7 | 495 (22.2) | 8 | 399 (51.9) | 5 | 416 (20.3) | 7 | 424 (33.7) | 8 | 456 (22.5) | 12 |
|  | DRvl | 620 (52.0) | 8 | 578 (27.5) | 8 | 531 (38.7) | 5 | 550 (19.7) | 6 | 558 (28.3) | 7 | 556 (20.9) | 12 |
|  | DRd | 546 (59.0) | 7 | 539 (38.1) | 8 | 465 (29.0) | 5 | 479 (15.1) | 7 | 498 (31.9) | 8 | 501 (23.4) | 12 |

**Supplementary Table 5.** Cross-Sectional area of NeuN-positive Cells (μm^2^)

|  | Con | | Scz | | Bip | | Dep | | Suic | | Non-Suic | |
| --- | --- | --- | --- | --- | --- | --- | --- | --- | --- | --- | --- | --- |
| Level | Mean (SEM) | N | Mean (SEM) | N | Mean (SEM) | N | Mean (SEM) | N | Mean (SEM) | N | Mean (SEM) | N |
| 1 | 306 (14) | 8 | 336 (34) | 8 | 274 (8) | 10 | 334 (20) | 7 | 315 (19) | 11 | 307 (19) | 14 |
| 2 | 412 (24) | 10 | 402 (39) | 6 | 413 (37) | 6 | 369 (16) | 7 | 400 (25) | 9 | 387 (26) | 10 |
| 3 | 464 (29) | 7 | 475 (23) | 6 | 445 (40) | 5 | 399 (23) | 6 | 417 (20) | 7 | 455 (25) | 10 |

NeuN cell area correlated with PH8 area (r=.32, p<.01) and brain weight (Level 2 r=.38 p<.05; Level 3 r=.54 p<.01).

**Supplementary Table 6.** Cross-Sectional Area of NeuN-positive Cells (μm^2^) by Subnucleus

|  | | Con | | Scz | | Bip | | Dep | | Suic | | Non-Suic | |
| --- | --- | --- | --- | --- | --- | --- | --- | --- | --- | --- | --- | --- | --- |
| Level | Nucleus | Mean (SEM) | N | Mean (SEM) | N | Mean (SEM) | N | Mean (SEM) | N | Mean (SEM) | N | Mean (SEM) | N |
| 2 | DRif | 423 (32.6) | 10 | 475 (69.9) | 5 | 482 (41.1) | 6 | 402 (30.2) | 7 | 487 (28.0) | 8 | 418 (40.5) | 10 |
|  | DRv | 458 (37.6) | 10 | 472 (48.9) | 6 | 452 (38.1) | 6 | 447 (42.7) | 7 | 423 (26.4) | 9 | 487 (37.1) | 10 |
|  | DRd | 387 (20.4) | 10 | 351 (38.3) | 6 | 389 (54.4) | 6 | 336 (13.4) | 7 | 379 (34.3) | 9 | 338 (25.4) | 10 |
| 3 | DRif | 437 (42.3) | 8 | 499 (35.4) | 6 | 485 (43.1) | 5 | 404 (21.4) | 6 | 459 (35.9) | 8 | 463 (25.5) | 9 |
|  | DRv | 453 (26.5) | 7 | 502 (30.0) | 6 | 450 (79.5) | 5 | 400 (24.5) | 7 | 415 (41.0) | 8 | 474 (33.8) | 10 |
|  | DRvl | 544 (30.0) | 8 | 611 (35.4) | 6 | 575 (49.8) | 5 | 534 (33.2) | 7 | 566 (33.6) | 8 | 575 (31.6) | 10 |
|  | DRd | 426 (32.9) | 7 | 415 (40.9) | 6 | 375 (20.9) | 5 | 342 (22.2) | 7 | 351 (13.5) | 8 | 395 (29.4) | 10 |

**Supplementary Table 7.** 5-HT_1A_R mRNA Emulsion-Dipped In Situ Hybridization: Cellular Grain Reflectance (OD) by Subnucleus

|  | | Con | | Scz | | Bip | | Dep | | Suic | | Non-Suic | |
| --- | --- | --- | --- | --- | --- | --- | --- | --- | --- | --- | --- | --- | --- |
| Level | Nucleus | Mean (SEM) | N | Mean (SEM) | N | Mean (SEM) | N | Mean (SEM) | N | Mean (SEM) | N | Mean (SEM) | N |
| 2 | DRif | .229 (.0790) | 11 | .150 (.0392)^a^ | 7 | .213 (.0308) | 6 | .192 (.0213) | 9 | .195 (.0294) | 11 | .175 (.0195) | 11 |
|  | DRv | .239 (.0093) | 11 | .158 (.0298)^b^ | 7 | .211 (.0389) | 6 | .207 (.0142) | 9 | .218 (.0194) | 11 | .167 (.0220) | 11 |
|  | DRd | .219 (.0202) | 11 | .184 (.0363) | 7 | .180 (.0341) | 6 | .203 (.0187) | 9 | .219 (.0170) | 11 | .163 (.0250) | 11 |
| 3 | DRif | .234 (.0159) | 8 | .198 (.0412) | 7 | .218 (.0542) | 6 | .198 (.0320) | 7 | .244 (.0361) | 9 | .172 (.0278) | 11 |
|  | DRv | .226 (.0173) | 8 | .234 (.0440) | 7 | .165 (.0384) | 6 | .193 (.0281) | 7 | .235 (.0405) | 9 | .169 (.0178) | 11 |
|  | DRvl | .205 (.0214) | 8 | .219 (.0535) | 7 | .182 (.0361) | 6 | .212(.0263) | 7 | .245 (.0381) | 9 | .173 (.0240) | 11 |
|  | DRd | .217 (.0126) | 8 | .205 (.0419) | 7 | .143 (.0449) | 6 | .205 (.0300) | 7 | .215 (.0410) | 9 | .164 (.0223) | 11 |

1. planned contrast scz<con p<.05 (Fig S7).
2. WMW scz<con p<.05 (Fig S8).
3. ANOVA p<.05; planned contrast suic>non-suic p<.05.

However, all analyses became non-significant when freezer storage time, post mortem interval and pH were included as covariates.

**Supplementary Table 8.** Estimated number of PH8-positive cells in a single 14μm section

|  | Con | | Scz | | Bip | | Dep | | Suic | | Non-Suic | |
| --- | --- | --- | --- | --- | --- | --- | --- | --- | --- | --- | --- | --- |
| Level | Mean (SEM) | N | Mean (SEM) | N | Mean (SEM) | N | Mean (SEM) | N | Mean (SEM) | N | Mean (SEM) | N |
| 1 | 74.3 (22.6) | 10 | 106 (23.5) | 8 | 70.5 (19.2) | 11 | 92.3 (26.5) | 9 | 124 (22.8)^a^ | 12 | 61.7 (11.5) | 16 |
| 2 | 372 (26.3) | 11 | 361 (37.7) | 7 | 409 (45.5) | 7 | 369 (46.7) | 8 | 406 (29.8) | 10 | 356 (37.4) | 12 |
| 3 | 415 (22.1) | 7 | 459 (37.1) | 8 | 466 (39.7) | 5 | 405 (17.1) | 7 | 431 (27.1) | 8 | 449 (26.6) | 12 |

1. KW ANOVA p<0.05; WMW suic>con p<.05.

**Supplementary Table 9.** Estimated number of NeuN-positive cells in a single 14μm section

|  | Con | | Scz | | Bip | | Dep | | Suic | | Non-Suic | |
| --- | --- | --- | --- | --- | --- | --- | --- | --- | --- | --- | --- | --- |
| Level | Mean (SEM) | N | Mean (SEM) | N | Mean (SEM) | N | Mean (SEM) | N | Mean (SEM) | N | Mean (SEM) | N |
| 1 | 109 (17.2) | 8 | 146 (37.5) | 8 | 136 (38.9) | 11 | 171 (38.3) | 7 | 206 (36.9)^a^ | 12 | 99.0 (17.9) | 14 |
| 2 | 471 (38.6) | 10 | 367 (102) | 5 | 446 (66.2) | 6 | 428 (67.2) | 7 | 423 (48.8) | 8 | 412 (67.8) | 10 |
| 3 | 442 (27.2) | 7 | 490 (62.3) | 6 | 418 (80.9) | 5 | 418 (53.0) | 5 | 480 (73.7) | 7 | 417 (33.2) | 9 |

1. KW ANOVA p<.05; WMW suic>non-suic p<.05.

**Supplementary Figures**

**
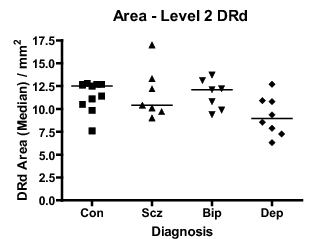
**

**Supplementary Figure 1.** Area of DRd at Level 2.

**
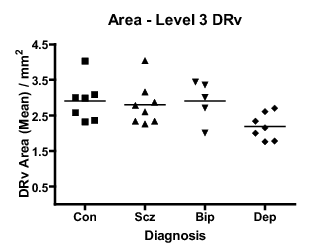
**

**Supplementary Figure 2.** Area of DRv at Level 3.


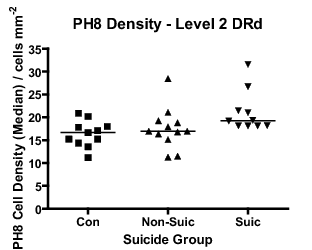


**Supplementary Figure 3.** PH8 cell density in DRd at Level 3.


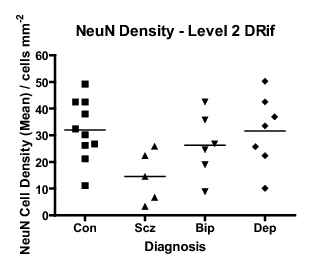


**Supplementary Figure 4.** NeuN cell density in DRif at Level 2.


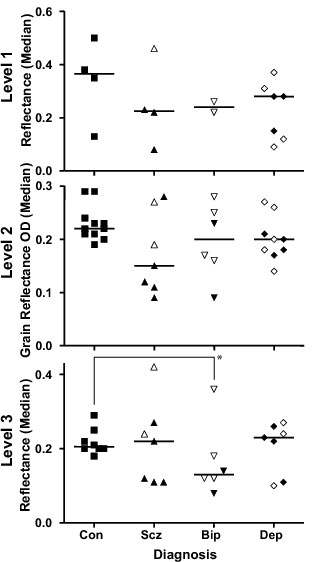
**Supplementary Figure 5.** 5-HT_1A_R mRNA ISHH Dipped Grain Reflectance OD (Per Cell).

As discussed in the text, there was a difference between groups at Level 3 (WMW bip<con p<0.05) but this became non-significant when freezer storage time, post mortem interval and and pH, each of which correlated with cellular 5-HT1AR mRNA, were included as covariates.


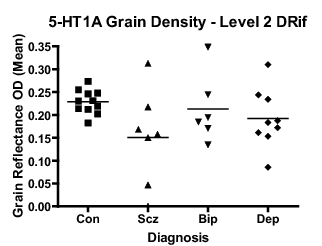
**Supplementary Figure 6**. 5-HT_1A_R mRNA Cellular Grain Reflectance OD in DRif at Level 2.


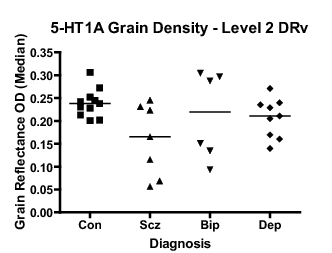
**Supplementary Figure 7**. 5-HT_1A_R mRNA Ceullar Grain Reflectance OD in DRv at Level 2.
